# Development and psychometric validation of the Pandemic Risk Perception Scale in healthcare professionals

**DOI:** 10.3389/fpsyg.2026.1878684

**Published:** 2026-10-12

**Authors:** Maria Carmen Badía, Rafael Alarcón, Javier Trillo, Jesús Miranda-Páez

**Affiliations:** 1 University of Malaga, Málaga, Spain; 2 Hospital Comarcal Santa Ana, Granada, Spain; 3 Department of Psychobiology and Behavioral Sciences Methodology, University of Malaga, Málaga, Spain; 4 Strategic Chair of Security, Emergencies and Disasters, University of Malaga, Málaga, Spain

**Keywords:** confirmatory factor analysis, COVID-19, healthcare professionals, risk perception, validity

## Abstract

**Introduction:**

Perceived pandemic-related risk may influence emotional responses, preventive behaviors, and psychological adjustment among healthcare professionals working in high-risk clinical settings. This study aimed to develop and evaluate the psychometric properties of the Pandemic Risk Perception Scale (PRPS) in healthcare professionals.

**Methods:**

A cross-sectional psychometric study was conducted with 405 healthcare professionals aged 22–65 years working in emergency and critical care settings in southern Spain. The PRPS was developed from an initial pool of 23 items and reduced to 12 items based on item analysis and exploratory examination of its dimensional structure. Its factor structure was subsequently examined using confirmatory factor analysis. Internal consistency was assessed using McDonald’s omega. Evidence of validity based on relationships with other variables was examined through correlations with depressive symptoms, anxiety symptoms, and psychological wellbeing.

**Results:**

Confirmatory factor analysis supported a unidimensional structure, with satisfactory overall model fit (χ^2^ = 168.31, df = 54, CFI = 0.99, TLI = 0.99, RMSEA = 0.07, 90% CI [0.06, 0.08]). Standardized factor loadings ranged from 0.56 to 0.92, and McDonald’s omega was 0.95. PRPS scores were positively associated with depressive symptoms (*r* = 0.44, *p* < 0.001) and negatively associated with psychological wellbeing (*r* = −0.22, *p* < 0.001). The association with generalized anxiety symptoms was small and not statistically significant (*r* = 0.069, *p* = 0.164).

**Conclusion:**

The findings provide preliminary evidence supporting the PRPS as a brief and reliable measure of perceived pandemic-related risk among healthcare professionals. Its associations with depressive symptoms and psychological wellbeing provide additional evidence of validity based on relationships with other variables. Further validation in independent and diverse samples is needed before extending its use to other populations, healthcare systems, or future public health emergencies.

## 1 Introduction

The coronavirus disease 2019 (COVID-19) pandemic, declared by the World Health Organization (WHO) in March 2020, represented an unprecedented global health emergency with substantial physical, social, and psychological consequences worldwide (Ferrer, 2020; Maguiña Vargas et al., 2020). Beyond its impact on physical health, the pandemic was associated with increased levels of anxiety, depression, stress, and sleep disturbances in both the general population and healthcare professionals (Hernández, 2020; Huarcaya-Victoria, 2020; Xiong et al., 2020; Peñafiel-León et al., 2021; Molina et al., 2020).

Healthcare professionals were among the groups most exposed to the challenges posed by the pandemic. Throughout successive waves of COVID-19, they faced high workloads, prolonged uncertainty, direct or potential exposure to infection, concerns about transmitting the virus to others, shortages of personal protective equipment, and emotionally demanding working conditions. These circumstances were particularly relevant for professionals providing direct patient care and were associated with substantial psychological burden and poorer mental health and wellbeing (Ferrán and Barrientos-Trigo, 2021; Santamaría et al., 2021; De Juan Pérez and Seguí Crespo, 2021; Danet, 2021; Rodríguez Zambrano and Elizabeth, 2020).

Within this context, risk perception represents an important psychological construct. It refers to the subjective evaluation individuals make regarding the probability of being exposed to a hazard and the potential consequences associated with that exposure (Acosta-Quiroz and Iglesias-Osores, 2023; Cori et al., 2020; Short, 1984; Slovic and Weber, 2013). In the context of infectious disease outbreaks, risk perception may influence preventive behaviors, decision-making, emotional responses, and adherence to protective measures. Previous research has suggested that perceived vulnerability, fear, perceived disease severity, knowledge, and confidence may contribute to how individuals evaluate health-related risks during public health emergencies (Ceberio et al., 2022; Casales, 2020; Restrepo, 2016; Pérez de Celis-Herrero and Cavazos-Arroyo, 2021).

For healthcare professionals, pandemic-related risk perception may involve dimensions that extend beyond general concerns about infection. Their perceptions may be influenced by direct occupational exposure to patients, responsibility for patient care, perceived vulnerability to infection, concerns about transmitting the disease to family members or colleagues, and the demands associated with working in high-pressure clinical environments. Consequently, assessing pandemic-related risk perception specifically among healthcare professionals may provide information that is not adequately captured by measures developed for the general population (Gerhold, 2020; Sandín et al., 2021; Shechter et al., 2020).

Although risk perception has been widely studied during the COVID-19 pandemic, many available instruments have been developed or evaluated primarily in samples from the general population or university students. Relatively few measures have been specifically designed to assess pandemic-related risk perception among healthcare professionals, despite their distinctive occupational exposure and responsibilities (Muñiz and Fonseca-Pedrero, 2019; Zeladita-Huamán et al., 2023). The availability of a population-specific instrument may therefore facilitate the assessment of perceived pandemic-related risk and its relationship with relevant psychological outcomes in healthcare settings.

Accordingly, the Pandemic Risk Perception Scale (PRPS) was developed to assess healthcare professionals’ perceived pandemic-related risk in the occupational context. The scale focuses on aspects of risk appraisal related to workplace information and communication, organizational support, protective protocols, and perceived safety when caring for patients with COVID-19. Importantly, PRPS scores are conceptualized as reflecting a continuous level of perceived risk, with higher scores indicating greater perceived risk rather than representing diagnostic or categorical classifications. The aim of the present study was therefore to examine the psychometric properties of the PRPS in healthcare professionals, including evidence of validity based on its internal structure, reliability, and associations with theoretically relevant variables, specifically depressive symptoms and psychological wellbeing.

## 2 Methods

### 2.1 Sample

A total of 405 healthcare professionals participated in this study, including 74.3% women and 25.7% men, with ages ranging from 22 to 65 years (*M* = 40.00, SD = 10.57).

Participants were recruited from hospital emergency departments, out-of-hospital emergency services, intensive care units, and other clinical units involved in the care of patients with COVID-19 in southern Spain. A non-probabilistic convenience sampling method was used.

Inclusion criteria were: (a) being at least 18 years old, and (b) working as a healthcare professional in clinical settings involved in the care of patients with COVID-19. Exclusion criteria were: (a) not meeting inclusion criteria, and (b) not providing informed consent.

### 2.2 Measures

Pandemic Risk Perception Scale (PRPS): The PRPS is an *ad hoc* questionnaire developed to assess healthcare professionals’ perceived pandemic-related risk in the occupational context. The scale was developed as part of a previous pilot study. The initial version of the questionnaire was developed through a consensus-based process involving an interdisciplinary expert panel comprising four professionals: two psychologists with expertise in assessment and psychometrics, and two nursing professionals (one female nurse and one male nurse) with clinical experience in high-risk contexts during the pandemic. This interdisciplinary panel generated a preliminary pool of 23 items covering the conceptual domain of pandemic-related risk in healthcare settings. The items were designed to assess aspects of risk appraisal relevant to the occupational context, including the adequacy and clarity of protective information, workplace communication, organizational support, protective protocols, and perceived safety when caring for patients with COVID-19.

Following the initial administration, items were evaluated according to their discriminatory value and corrected item-total correlations (corrected homogeneity index). Based on these analyses, items showing inadequate discriminatory properties or weak corrected item-total correlations were removed, resulting in a final 12-item version of the PRPS. The 12 items comprising the final version of the PRPS are presented in Table 1.

**Table 1:** Items of the Pandemic Risk Perception Scale (PRPS).

Item	PRPS item
1	My workplace adequately informs me about how to protect myself against COVID-19
2	I feel supported by my supervisors when carrying out my work with patients with COVID-19
3	The information provided by my workplace about measures to protect against COVID-19 is scarce or confusing
4	My workplace is concerned about my safety and protection against COVID-19.
5	The communication regarding COVID-19 used by my workplace with professionals is insufficient
6	My workplace is concerned that I have adequate knowledge of how to protect myself against COVID-19
7	My workplace adequately ensures compliance with protocols and regulations to protect me against COVID-19
8	I feel uninformed and confused by the guidelines provided by my workplace on how to act and protect myself when caring for patients with COVID-19
9	The COVID-19 protocol established by my workplace for professionals caring for suspected COVID-19 cases is adequate and sufficient
10	I feel safe at work regarding the measures implemented to prevent COVID-19 infection.
11	At times, I feel abandoned when working with patients with COVID-19
12	The communication process used by my workplace is adequate for informing professionals and conveying the instructions we should follow regarding COVID-19

Each item is rated on a 5-point Likert-type scale ranging from 0 (strongly disagree) to 4 (strongly agree). Total scores range from 0 to 48, with higher scores indicating greater perceived pandemic-related risk. The PRPS is conceptualized as a continuous measure of perceived risk, and no diagnostic or categorical cut-off scores were established in the present study.

Patient Health Questionnaire (PHQ-9): The PHQ-9 is a self-report instrument assessing depressive symptoms over the previous 2 weeks. It consists of nine items rated on a 4-point Likert scale (0–3), with total scores ranging from 0 to 27. Higher scores indicate greater severity of depressive symptoms. The scale has shown good psychometric properties in Spanish populations (Patel et al., 2019; Diez-Quevedo et al., 2001).

Generalized Anxiety Disorder Scale (GAD-7): The GAD-7 assesses generalized anxiety symptoms over the previous two weeks. It includes seven items rated on a 4-point Likert scale (0–3), with total scores ranging from 0 to 21. Higher scores indicate greater anxiety severity. The scale has demonstrated good reliability and validity in previous studies (Johnson et al., 2019).

Psychological Wellbeing Scale (PWBS): The reduced Spanish version of the PWBS, based on Ryff’s model, was used to assess psychological wellbeing. It consists of 29 items rated on a 6-point Likert scale (Ferrer, 2020; Maguiña Vargas et al., 2020; Hernández, 2020; Huarcaya-Victoria, 2020; Xiong et al., 2020; Peñafiel-León et al., 2021) and evaluates six dimensions: self-acceptance, positive relationships, autonomy, environmental mastery, purpose in life, and personal growth. The scale has shown adequate reliability and construct validity in Spanish samples (Ryff, 1989; Van Dierendonck, 2004; Rosseel, 2012; Díaz et al., 2006).

### 2.3 Procedure

The study was conducted in accordance with the ethical principles established in the Declaration of Helsinki. Ethical approval was obtained from the Ethics Committee of the University of Malaga (approval code: 41-2021-H; approval date: 6 August 2021).

Data were collected between May and September 2021 using an anonymous online questionnaire administered to healthcare professionals working in hospital emergency departments, out-of-hospital emergency services, intensive care units, and other clinical settings involved in the care of patients with COVID-19 in southern Spain. Before accessing the questionnaire, participants were provided with information about the study and were required to provide informed consent through a mandatory digital consent form. Participants who did not provide consent were not permitted to proceed with the questionnaire.

Participation was voluntary, confidential, and unpaid. No personally identifying information was collected. Data were handled anonymously and securely, and confidentiality and data protection were maintained throughout the research process in accordance with applicable ethical and legal requirements.

### 2.4 Data analysis

Descriptive statistics were calculated to summarize the sociodemographic and occupational characteristics of the participants.

The dimensionality and internal structure of the PRPS were examined using an exploratory procedure followed by confirmatory factor analysis (CFA). First, an exploratory analysis was conducted in the pilot sample (*n* = 99). Sampling adequacy was assessed using the Kaiser–Meyer–Olkin (KMO) measure and Bartlett’s test of sphericity. The number of dimensions was examined based on eigenvalues, the scree plot, and item/component loadings. A principal component analysis (PCA) was conducted as an exploratory data-reduction procedure to examine the dimensional pattern of the items and determine whether a dominant component was present before the CFA. A KMO value ≥ 0.80 was considered indicative of adequate sampling adequacy, and item/component loadings ≥ 0.40 were considered acceptable.

The same exploratory procedure was subsequently conducted in the final sample (*n* = 405) as an additional exploratory examination of the dimensional structure before conducting the CFA. Because the exploratory analysis and CFA were conducted in the same final sample, the exploratory findings were considered preliminary and were not treated as an independent replication of the pilot findings.

Evidence of validity based on internal structure was subsequently examined using CFA in the final sample (*n* = 405). A one-factor model was specified, with the 12 PRPS items loading on a single latent factor representing pandemic-related risk perception. The CFA was conducted using the lavaan package in R (Rosseel, 2012).

Model fit was evaluated using the chi-square statistic (χ^2^), comparative fit index (CFI), Tucker–Lewis index (TLI), and root mean square error of approximation (RMSEA), together with its 90% confidence interval. Following established recommendations, CFI and TLI values ≥ 0.95 were considered indicative of good model fit, whereas RMSEA values ≤ 0.08 were considered acceptable and values ≤ 0.06 indicative of close fit (Bentler, 1990; Bentler and Bonett, 1980; Browne and Cudeck, 1992; Hu and Bentler, 1999).

Internal consistency reliability was assessed using McDonald’s omega (ω), with values ≥ 0.70 considered acceptable. Item performance was examined using corrected item-total correlations, with coefficients ≥ 0.30 considered acceptable.

Evidence of validity based on relationships with other variables was examined using Pearson correlation coefficients between PRPS scores and scores on the Patient Health Questionnaire-9 (PHQ-9), Generalized Anxiety Disorder-7 (GAD-7), and Psychological Wellbeing Scale (PWBS). Positive associations were hypothesized between PRPS scores and depressive and anxiety symptoms, whereas a negative association was hypothesized between PRPS scores and psychological wellbeing. The magnitude of the correlations was interpreted according to Cohen’s criteria, with absolute correlation coefficients of approximately 0.10, 0.30, and 0.50 representing small, moderate, and large associations, respectively (Cohen, 1988).

Statistical significance was set at *p* < 0.05 for all analyses.

## 3 Results

### 3.1 Descriptive analysis

Table 2 presents the sociodemographic and occupational characteristics of the participants, including professional category, living arrangements, cohabitation with individuals aged over 60 years, and previous COVID-19 infection status.

**Table 2:** Sample characteristics.

Variable	Percentage
Type of healthcare professional	
Doctor	12.6
Nurse	63.2
Nurse’s auxiliary	21.0
Emergency technician	3.2
Living alone	
Yes	14.3
No	85.7
Living with a person older than 60 years	
Yes	14.6
No	85.4
You have been infected with COVID-19	
Yes	19.3
No	80.7

### 3.2 Evidence of validity based on internal structure

The confirmatory factor analysis supported a unidimensional structure of the Pandemic Risk Perception Scale (PRPS). Model fit indices indicated satisfactory overall fit: χ^2^(54) = 168.31, CFI = 0.99, TLI = 0.99, and RMSEA = 0.07 (90% CI [0.06, 0.08]).

All standardized factor loadings were statistically significant and ranged from 0.56 to 0.92 (Table 3), providing further support for the proposed single-factor structure.

**Table 3:** Fit indices for the unidimensional structure of the Pandemic Risk Perception Scale (PRPS).

Model	χ	df	CFI	NNFI	RMSEA (90% CI)
Unidimensional model	168.31	54	0.99	0.99	0.07 [0.06, 0.08]

χ^2^, chi-square statistic; df, degrees of freedom; CFI, comparative fit index; NNFI, non-normed fit index; RMSEA, root mean square error of approximation; CI, confidence interval.

### 3.3 Reliability and item analysis

The PRPS demonstrated high internal consistency, with a McDonald’s omega coefficient of ω = 0.95. Corrected item-total correlations ranged from 0.51 to 0.85, indicating adequate item discrimination. Standardized factor loadings ranged from 0.56 to 0.92, with all loadings statistically significant (*p* < 0.001). Item-level factor loadings and corrected item-total correlations are presented in Table 4.

**Table 4:** Standardized factor loadings for the unidimensional structure of the COVID-19 Pandemic Risk Perception Scale and corrected item-total correlation.

Item	Factor loading	Corrected item-total correlation
1	0.91	0.85
2	0.88	0.82
3	0.56	0.51
4	0.92	0.84
5	0.72	0.67
6	0.91	0.84
7	0.90	0.83
8	0.83	0.78
9	0.83	0.76
10	0.87	0.81
11	0.78	0.72
12	0.85	0.79

All standardized factor loadings were statistically significant (*p* < 0.001).

### 3.4 Validity evidence based on relationships with other variables

PRPS scores showed a moderate positive correlation with depressive symptomatology assessed using the Patient Health Questionnaire-9 (PHQ-9) (*r* = 0.435, *p* < 0.001), indicating that higher perceived pandemic risk was associated with greater depressive symptomatology. In contrast, the correlation between PRPS scores and anxiety symptoms assessed using the Generalized Anxiety Disorder-7 (GAD-7) was small and not statistically significant (*r* = 0.069, *p* = 0.164). PRPS scores were also negatively correlated with psychological wellbeing assessed using the Reduced Spanish Version of the Psychological Wellbeing Scale (PWBS) (*r* = −0.22, *p* < 0.001), indicating that higher perceived risk was associated with lower psychological wellbeing.

## 4 Discusion

The aim of this study was to examine the psychometric properties of the Pandemic Risk Perception Scale (PRPS) in healthcare professionals working in emergency and critical care settings during the COVID-19 pandemic. Overall, the findings provide preliminary evidence supporting the reliability and validity of PRPS scores in this specific professional and occupational context. The scale demonstrated a coherent unidimensional structure, high internal consistency, adequate item performance, and theoretically relevant associations with indicators of psychological functioning. Data collection took place between May and September 2021, during a period of sustained clinical pressure associated with the COVID-19 pandemic in Spain. Healthcare professionals working in emergency departments, prehospital emergency services, intensive care units, and other services involved in the care of patients with COVID-19 were exposed to persistent uncertainty, risk of infection, high workload, and the emotional demands associated with caring for seriously ill patients. These contextual characteristics are relevant when interpreting pandemic-related risk perception in healthcare settings, as occupational risk may be shaped by workplace exposure and by the availability, clarity, and adequacy of information, protective measures, organizational support, and safety protocols. These contextual factors provide a rationale for considering pandemic-related risk perception within the specific occupational environment in which healthcare professionals provide patient care.

The evidence regarding the internal structure of the PRPS was supported by an exploratory phase followed by confirmatory factor analysis. The exploratory analyses supported a unidimensional structure, which was subsequently tested through CFA. The one-factor CFA model showed satisfactory fit, with χ^2^(54) = 168.31, CFI = 0.99, TLI = 0.99, and RMSEA = 0.07 (90% CI [0.06, 0.08]). Standardized factor loadings ranged from 0.56 to 0.92, indicating that all items contributed meaningfully to the measurement of the underlying construct.

The PRPS also demonstrated high internal consistency, with a McDonald’s omega coefficient of ω = 0.95. Together with the adequate item-level performance, these findings suggest that the retained items show substantial coherence in measuring perceived pandemic risk. Importantly, high internal consistency should not be interpreted as evidence of unidimensionality by itself; rather, the interpretation of the PRPS as a unidimensional measure is supported by converging evidence from the exploratory analyses and CFA.

The unidimensional structure identified in the present study is consistent with research in which COVID-19 risk perception has been conceptualized as a relatively global construct, although existing instruments differ considerably in their content and dimensional structure. For example, the COVID-19 Risk Perception Scale (CoRP) was developed and subsequently supported as a one-factor measure in Italian community samples, whereas other instruments developed for healthcare workers have incorporated more specific domains, including occupational exposure, personal safety, psychosocial risk, and organizational conditions. Thus, the present findings suggest that a brief, unidimensional measure of perceived pandemic-related risk can be psychometrically useful in healthcare professionals, while recognizing that the broader construct may encompass several related occupational and psychological processes.

Accordingly, the unidimensional structure of the PRPS should be understood as a measurement property of the present instrument rather than as evidence that pandemic risk perception is inherently unidimensional across populations, settings, or public health emergencies.

Evidence of validity based on relationships with other variables was also observed. PRPS scores showed a moderate positive correlation with depressive symptoms assessed using the Patient Health Questionnaire-9 (PHQ-9) (*r* = 0.435, *p* < 0.001). Higher perceived pandemic risk was therefore associated with greater depressive symptomatology. PRPS scores were also negatively correlated with psychological wellbeing assessed using the Reduced Spanish Version of the Psychological Wellbeing Scale (PWBS) (*r* = −0.22, *p* < 0.001), indicating that greater perceived risk was associated with lower psychological wellbeing. These findings are consistent with previous research describing the psychological burden experienced by healthcare professionals during the COVID-19 pandemic, including stress, emotional exhaustion, anxiety, and burnout (Cohen, 1988; Lozano-Vargas, 2020; Muñoz-Fernández et al., 2020; Buitrago Ramírez et al., 2022; Lozano-Vargas, 2021; Campoy Aranda et al., 2023).

In contrast, the association between PRPS scores and anxiety symptoms assessed using the Generalized Anxiety Disorder-7 (GAD-7) was small and not statistically significant (*r* = 0.069, *p* = 0.164). This finding is relevant because it suggests that PRPS scores should not simply be interpreted as a direct measure of generalized anxiety. At the same time, the significant associations with depressive symptoms and psychological wellbeing indicate that perceived pandemic risk is related to broader aspects of psychological functioning. These findings should be interpreted as evidence based on relationships with other variables rather than as definitive evidence of discriminant validity, which would require a more comprehensive assessment involving theoretically distinct constructs and appropriate psychometric analyses.

The observed associations may be understood from a cognitive appraisal perspective, whereby perceptions of threat can influence emotional and behavioral responses to stressful circumstances. However, the cross-sectional design of the present study prevents conclusions regarding the direction of these relationships. Higher perceived risk may contribute to psychological distress, but professionals experiencing greater psychological distress may also perceive occupational threats more negatively. Longitudinal studies are therefore needed to clarify the temporal and potentially bidirectional relationships between perceived risk and psychological functioning.

An important contribution of the present study concerns the population and occupational context for which the PRPS was developed. Previous COVID-19 risk-perception instruments have been developed or evaluated in different populations, including community samples, students, and healthcare professionals, and they differ substantially in their conceptualization and measurement of risk. For example, the CoRP was developed for the general population, whereas other instruments have examined risk perception among nursing students or healthcare workers and have incorporated broader domains such as preventive behavior, occupational exposure, psychosocial risk, and organizational conditions.

Unlike instruments designed primarily to assess general perceptions of COVID-19 risk, the PRPS was developed specifically for healthcare professionals working in settings characterized by direct exposure to patients with COVID-19. The item content reflects the occupational context in which pandemic-related risk was experienced, with particular emphasis on access to protective information, clarity of workplace guidance, organizational support, adequacy of protective protocols, and perceived safety when caring for patients with COVID-19. This population-specific focus is relevant because perceived risk in healthcare settings may be influenced by contextual and organizational conditions that shape professionals’ perceptions of their ability to protect themselves while providing patient care. Accordingly, the PRPS should be interpreted as a focused measure of perceived pandemic-related risk within the occupational context examined, rather than as a comprehensive assessment of all dimensions that may contribute to risk perception, such as perceived probability of infection or perceived severity of illness.

The PRPS was also designed as a brief instrument focused specifically on perceived pandemic-related risk within the occupational context, rather than combining this construct with broader domains such as preventive knowledge, coping behaviors, or psychological symptoms. Its concise format may facilitate administration in clinical and occupational settings where assessment time is limited. Nevertheless, its brevity should not be interpreted as evidence that pandemic risk perception is a simple construct; rather, the scale provides a focused measure of a specific aspect of risk perception within the occupational context examined.

From an applied perspective, the PRPS may be useful in research examining how healthcare professionals perceive occupational risk during public health emergencies and how such perceptions relate to psychological functioning. It may also facilitate the monitoring of perceived risk in occupational and research contexts, particularly when brief assessment is desirable.

However, the current findings do not establish that the PRPS can be used as a clinical screening instrument or that it can identify healthcare professionals who will subsequently develop psychological problems. The PRPS should therefore be interpreted as a continuous measure of perceived pandemic risk rather than as a diagnostic or categorical screening tool. Its clinical or screening utility would require additional evidence, including criterion-related validity, clinically meaningful cut-off scores, sensitivity and specificity analyses, and longitudinal validation.

Several limitations should be acknowledged. First, all measures were based on self-report, which may introduce response bias and common method variance. Second, participants were recruited using non-probabilistic convenience sampling, limiting the generalizability of the findings to other healthcare populations, institutions, and geographical contexts. Third, the cross-sectional design prevents causal conclusions regarding the relationships between perceived pandemic risk and psychological outcomes. Fourth, the study was conducted in the specific context of the COVID-19 pandemic and during a particular phase of the pandemic in Spain. Therefore, the extent to which PRPS scores generalize to other infectious disease outbreaks or future public health emergencies remains to be established. Fifth, the content of the final PRPS items primarily reflects organizational and occupational aspects of perceived pandemic-related risk, including the adequacy and clarity of information, workplace communication, organizational support, protective protocols, and perceived safety. Other dimensions that may form part of broader conceptualizations of risk perception, such as perceived susceptibility, perceived severity, or concerns about transmission to others, were not directly assessed. Therefore, the PRPS should be considered a focused measure of occupational pandemic-related risk perception rather than a comprehensive assessment of the broader risk-perception construct. Sixth, although the initial exploratory analysis was conducted in an independent pilot sample (*n* = 99), an additional exploratory PCA was conducted in the final sample (*n* = 405) before the CFA. Because the exploratory and confirmatory analyses in the final sample were based on the same participants, the possibility of capitalization on chance cannot be completely excluded. Therefore, the factor structure should be considered preliminary and requires confirmation in independent samples. Finally, test–retest reliability was not assessed in the present study, and the temporal stability of PRPS scores remains unknown.

Further psychometric research should replicate the factor structure in independent samples and examine the stability and invariance of the measurement model across professional groups, healthcare settings, sex, age, and different cultural or healthcare-system contexts. Additional evidence of discriminant and criterion-related validity would also strengthen the psychometric evaluation of the instrument. Test–retest studies should also examine the temporal stability of PRPS scores when the underlying level of perceived risk is expected to remain relatively unchanged. Longitudinal studies would be particularly valuable for examining whether changes in perceived risk precede changes in psychological distress and wellbeing.

Future research should also investigate potential moderators and mechanisms underlying the relationship between perceived pandemic risk and psychological outcomes, including resilience, coping strategies, organizational support, previous exposure to infectious diseases, and intensity of occupational exposure. Such research could help clarify under which circumstances perceived risk is associated with psychological distress and when it may instead be related to adaptive preventive behaviors.

Overall, the findings provide preliminary evidence supporting the PRPS as a brief, reliable, and psychometrically coherent measure of perceived pandemic risk among healthcare professionals working in high-exposure clinical settings during the COVID-19 pandemic. The evidence from exploratory and confirmatory analyses supports its unidimensional structure, while its associations with depressive symptoms and psychological wellbeing provide additional evidence of validity based on relationships with other variables. Further validation in independent and diverse samples is needed before extending its use to other populations, healthcare systems, or future public health emergencies.

## Data Availability

The datasets generated and/or analysed during the current study are available in the repository: 555 https://dx.doi.org/10.24310/riuma.35195.
